# Erratum for Early‐stage Parkinson's disease: Abnormal nigrosome 1 and 2 revealed by a voxel wise analysis of neuromelanin‐sensitive MRI

**DOI:** 10.1002/hbm.25895

**Published:** 2022-04-26

Table 2 contains some typographical errors in the last two rows in the Healthy subjects column, which do not affect the results of the paper. The corrected table appears here.

**TABLE 2:** The ranges of contrast ratio (CR) values of the areas with significant group difference between IPD patients and healthy subjects on NM‐MRI

	IPD patients (n = 50)				Healthy subjects (n = 50)			
	Minimum	Maximum	Mean	SD	Minimum	Maximum	Mean	SD
Significantly different regions	.089556	.217321	.141522	.031067	.108020	.274834	.1834127	.0310916
Whole SNpc	.076808	.189195	.127170	.025358	.085074	.198957	.1396475	.0239943
